# On the Cost of Conforming to Reviewers’ Expectations and the Potential Benefit of Prediction Competitions

**DOI:** 10.3390/e28101044

**Published:** 2026-09-22

**Authors:** Ido Erev, Adi Tarabeih, Rachel Barkan

**Affiliations:** 1 Department of Psychology, The Ohio State University, Columbus, OH 43210, USA; erev.1@osu.edu; 2 Faculty of Data and Decision Sciences, The Technion—Israel Institute of Technology, Haifa 3200003, Israel; adi.tarabeih@campus.technion.ac.il; 3 Guilford Glazer Faculty of Management, Ben-Gurion University of the Negev, Beer-Sheva 8410501, Israel

**Keywords:** insufficient exploration, conformity, sequential convention game, paradigm shift, reliance on small samples

## Abstract

Although academic peer review offers many important benefits, it can also impede scientific exploration. For instance, when reviewers share restrictive working assumptions, researchers may be incentivized to conform to them, even when alternative conjectures could better advance scientific understanding. We suggest mitigating this problem by complementing peer review with open prediction competitions. We illustrate the feasibility of this approach with two competitions on predicting decisions under risk. For more than 70 years, influential research on choice behavior has focused on models assuming that decision makers trust descriptions of payoff distributions, evaluate each option in isolation, and assign subjective values and weights to outcomes and probabilities. In contrast, the best-performing models in two recent prediction competitions relied on markedly different assumptions. They assume that decision makers question the accuracy of stated descriptions, use them as cues to retrieve similar past experiences, and select the strategy that proved most successful in analogous situations. We propose that, when evaluating papers that refine existing models, journal editors consider the decision “acceptance conditional on organizing an appropriate prediction competition.” Such competitions would test whether alternative assumptions achieve superior predictive performance, encouraging creativity and theoretical innovation while grounding scientific evaluation in predictive success.

## 1. Introduction

Suboptimal decisions often reflect insufficient exploration. For example, the first author of the current paper still looks at the keyboard while typing. Although this habit slows him down and may contribute to back pain, he has not mastered touch-typing. He has attempted to learn touch-typing but has yet to discover a method that yields better outcomes for him than his familiar hunt-and-peck strategy. This example illustrates a broader tendency to persist with familiar behaviors rather than invest in alternatives that may ultimately prove superior.

Busemeyer and Myung [1] demonstrated a similar pattern in a simple experimental task involving repeated choices among alternatives located in a two-dimensional space. Their findings revealed a tendency to converge on local maxima with broad basins of attraction, a pattern that can be captured by models assuming hill-climbing processes.

Figure 1 demonstrates this pattern, summarizing an experiment reported by Erev and Haruvy [2] that compared two multi-alternative choice tasks. Both tasks involved choices among 400 alternatives (presented as a 20 × 20 matrix). Each alternative was associated with a single deterministic outcome, and participants received feedback about the obtained payoff after every choice. The two tasks differed only in the spatial arrangement of the 400 alternatives within the 20 × 20 matrix. The middle panel of Figure 1 presents a three-dimensional representation of the two matrices. As shown, both matrices contained two maxima: a local maximum yielding 32 points and a global maximum yielding 52 points per choice. In one condition, the global maximum (52) was surrounded by a wide basin of attraction (top graph). The other condition was created by swapping the locations of the two maxima, such that the local maximum (32) was associated with the wider basin of attraction (bottom graph). The right-hand panel of Figure 1 presents the proportion of maximizing choices in the two conditions. Consistent with the findings of Busemeyer and Myung [1], participants came closer to maximization when the global maximum was associated with the wider basin of attraction.

Inefficiencies of the type described above (that is, failures to converge on maximizing behavior) are often mitigated by social interaction. For example, it is reasonable to expect that observing other participants repeatedly earning 52 points by selecting a particular key would lead to conformity, encouraging additional participants to adopt the same strategy. Yet this is not always the case. In some settings, social interaction can instead aggravate the problem by reinforcing counterproductive conformity [3].

One experimental demonstration of the coexistence of positive and negative effects of social interaction is provided by Aboful, Erev, and Sulitzeanu-Kenan [4]. Their experimental task involved 100 repeated choice trials. In each trial, participants chose between two unmarked keys and received immediate feedback about both the obtained and forgone payoffs. The basic payoffs associated with both keys were either a gain of 7 or a loss of 5. However, for one key, the probability of a gain remained constant at *p* = 0.70 across all 100 trials. For the other key, the probability of a gain was *p* = 0.90 during the first 60 trials but declined to *p* = 0.50 during the final 40 trials.

In a control condition, participants performed the task individually. In a “conformity” condition, participants performed the task in groups of three, and the groups’ decisions were determined by majority rule. Importantly, disagreement with the majority was penalized: a participant who chose against the majority lost 2 points, while each member of the majority gained an additional 1 point. The results revealed that social interaction (i.e., the conformity condition) increased the maximization rate during the first 60 trials to 79%, compared with 63% in the control condition (i.e., individual decision-making). However, during the final 40 trials, the same social interaction that encouraged conformity reduced the maximization rate to 37% (i.e., conformity condition) compared with 54% in the control condition (i.e., individual decision making). Thus, social interaction was less effective in adapting to the change in probability.

The current paper extends the study of social interactions that reinforce conformity. We begin by demonstrating that when deviations from social convention reduce the probability of success, social interaction is detrimental, triggering behavior that resembles extreme and counterproductive risk aversion. In this demonstrative example, conformity-oriented incentives can lead groups to prefer a relatively safe gamble, offering an equal chance of winning 7 or 1 (expected value = 4), over a riskier yet superior gamble, offering an equal chance of winning 20 or 0 (expected value = 10).

We then draw an analogy to traditional academic review, raising the possibility that its procedures can produce similar conformity-based inefficiencies. Specifically, we propose that peer review may encourage researchers to favor safer research paradigms, even when riskier paradigms have greater potential to generate useful insights. We examine the feasibility of this hypothesis, and a potential way to address it, by considering the outcomes of recent choice prediction competitions designed to reduce the cost of building on less conventional working assumptions.

## 2. The Sequential Conformity Game

Consider a repeated choice task performed by one or more decision makers (hereafter, players). In each trial, one player (a randomly selected “acting player”) first chooses between a safe gamble that yields 7 or 1 points and a risky gamble that pays 20 or 0 points. If the number of players is larger than one, the choice of the acting player is compared with the choices of other players (past choices of two different players, if possible, and the very next choices in the case of the first two acting players). The other players are construed as overseers of the acting player. The probability that the acting player wins the higher payoff is 0.5 if his or her choice does not deviate from the choices of the overseeing players (i.e., conforming to a convention of the overseers). The probability of winning the higher payoff is reduced to 0, however, if at least one of the overseeing players has chosen the alternative option (i.e., no convention between overseers).

When the game is played sequentially with *full information*, rational risk-neutral players are expected to select Risk (i.e., the gamble between 20 and 0). This prediction is derived by backward induction. (Note: If the third acting player (mover) sees that the first two have chosen Risk, he or she should do the same. For that reason, if the second mover sees that the first has chosen Risk, he or she should select Risk too. Anticipating this reaction to his or her choice, the first mover should select Risk. The decisions of the other players are like the decision of the third mover: If all past players have chosen Risk, Risk is the best choice.) In contrast, however, for *n* > 1 players, with *limited information* such that players make their choices not knowing their serial position in the decision order, the interaction between the players becomes a coordination game with *at least two pure Nash equilibria:* a Pareto-efficient equilibrium in which the expected payoff is 10 (where all the players choose Risk), and an inefficient equilibrium in which the expected payoff is 4 (where all the players choose the safer gamble). (Note: Nash equilibrium is a profile of strategies from which no player can benefit from unilateral deviation. To see that a strategy profile in which all the players select Risk is an equilibrium, note that unilateral deviation reduces the expected payoff from 10 to 1. Similarly, a strategy profile in which all the players select Safe is an equilibrium, as unilateral deviation reduces the expected payoff from 4 to 0.)

The current analysis focuses on the latter situation in which players have limited information. They do not receive a description of the incentive structure and have no information about their position in the decision order. Rather, they must rely on feedback about the outcomes of their previous choices. Rational analysis cannot be used to derive clear analytical predictions in this setting: in the multi-equilibria coordination game, almost any behavioral pattern can be justified as rational under certain assumptions about players’ beliefs. To address this difficulty, we build on experimental research that highlights the predictive value of simulations of simple learning models. Figure 2 presents the predicted choice probabilities of players via simulations of three learning models (described in Appendix A) for this game with *n* = 1, 3, and 10 players. The results show that the simulations of all three models predict a large decrease in the probability of obtaining the maximal payoff when the number of players increases from 1 (where the game reduces from a coordination game to an individual choice task) to 3, followed by a smaller, diminishing decrease as the number of players exceeds 3.

## 3. Peer Review as a Problematic Coordination Game and the Potential Solution of Competition

Previous research [5,6,7,8,9,10,11,12,13] suggests that the incentive structure in certain academic communities shares significant similarities with the sequential conformity game described above. For example, Haubrock et al. [13] assert that for early career researchers “strategic conformity becomes a rational career path… suppressing creativity and critical thinking precisely at the stage when scientific independence could otherwise emerge”. One keystone of this conjecture builds on Kuhn’s analysis of the structure of scientific revolutions [14]. Kuhn’s analysis suggests that researchers can choose between distinct sets of working assumptions to guide their investigations. Some sets are relatively safe: they are likely to lead to publishable papers (this set includes the assumptions implied by Kuhn’s “normal science”). Other sets of working assumptions are riskier. Selecting assumptions of this type may result in high rewards, but it reduces the overall probability of positive outcomes and is less likely to lead to publishable papers (this set includes the possibility of leading to a paradigm shift, but it also includes many futile research directions).

A second keystone of the conjecture is that reviewers are likely to be more supportive of papers that share their working assumptions. This tendency may arise because it is easier to understand, evaluate, and appreciate papers related to one’s own research. The assumed reviewer bias may also reflect the fact that papers sharing reviewers’ working assumptions are more likely to cite the reviewers themselves. Recent research demonstrates that the probability of a positive review increases when reviewers are cited [15]. Moreover, reviewers are unlikely to favor assertions that question the value of their own previous research [5]. Research also shows that funding for risky research projects is less likely to be sustained [11], and that in response, researchers strategically adjust their research proposals, transforming their original ideas into predictable, short-term refined developments of established research [12].

Kuhn based his analysis on important historical examples. Consider the example of basic hygiene in medicine. For centuries, much of medicine operated under the assumption that disease came from “miasma” (bad air). This was treated more as a truism than as an assumption that could be challenged. Although direct contagion, the effects of contaminated tools, and the benefits of hand hygiene were observed often enough, physicians operating under this truism had little reason to attend to them [14].

The “miasma” framework delayed recognition of critical observations. For example, Ignaz Semmelweis showed in the 1840s that washing hands drastically reduced maternal deaths [16]. However, his findings were largely resisted because they did not fit dominant medical assumptions. Germ theory later explained what he had observed, and the miasma theory was eventually replaced by microbial explanations [14]. It is important to recognize, however, that, in typical research projects, researchers can choose among many risky courses of action and that many of them may be futile. Taking risks is not always optimal. Moreover, a leading theory may be more effective than proposed alternatives even when some of its predictions can be rejected.

Lakatos [17] proposed addressing this difficulty by comparing competing research programs and preferring those that continue to produce novel explanatory and predictive successes. Yet, as demonstrated by our sequential conformity game above, the problematic incentive structure may impair the effective comparisons suggested by Lakatos.

We propose that the barrier to effective comparison of research programs can be partially addressed using open prediction competitions [18,19,20,21,22,23,24,25,26]. Under this research methodology, a group of researchers organizes a competition focused on predicting a sample of future events from a well-defined space of events. For example, in experimental decision research [23,24,25,26], the organizers first run a training experiment that examines behavior in a wide set of tasks randomly drawn from the competition space. Next, the organizers publish the empirical findings and challenge other researchers to develop a model that accounts for these findings and can be used to predict choice behavior in a second “target” experiment. This “target” experiment examines behavior in a different sample of tasks randomly drawn from the competition space. Each proposed model is developed to best fit the given empirical observations and is then tested on its ability to predict the yet-unknown behaviors that would emerge in the new experimental tasks.

Importantly, the competing models are compared using an objective criterion, for example, the mean squared distance between predicted and observed choice rates. Thus, evaluation of the submitted models does not depend on subjective judgments by reviewers. The competition allows researchers to select their working assumptions freely. Model accuracy is the focus of evaluation, whereas assumptions are not evaluated: the competition aims to reduce the discrepancy between models’ predictions and the observed results. The winning model presents the minimal discrepancy and best predicts the observed results. Competitions of this type can therefore reduce pressure to conform to specific conventions and frameworks. Indeed, researchers can benefit from trying novel approaches in their models.

### 3.1. Numerical Demonstration

To illustrate the potential benefit of prediction competitions, consider a variant of the sequential conformity game described above. In this variant, the outcomes represent prediction accuracy scores, and the players are researchers choosing between two options (i.e., their sets of assumptions/model predictions). Assume there are *n* players and that each player must choose between a safe option, where the prediction accuracy score is equally likely to be 1 or 7, and a risky option, where the prediction accuracy score is equally likely to be 0 or 20. However, the payoff is obtained only if the player is not outperformed by another player. Consequently, this variant of the game has *only one equilibrium*, in which all players select the risky option (i.e., an equal chance of 0 or 20). Applying simulations of the three learning models above indicates that, opposite to the original sequential conformity game, in this variant the proportion of trials in which the maximal payoff (20) is obtained by at least one player increases with *n* (the number of players). Figure 3 presents the simulated predictions of these three learning models. It shows that changing the payoff rule to reward accuracy leads to rapid convergence to the single equilibrium. Importantly, maximization of prediction accuracy increases with *n*.

### 3.2. The Example of Decisions Under Uncertainty

The assertion that the incentive structure in some academic communities resembles that of the sequential conformity game described above leads to two nontrivial predictions. First, a scientific community may study a particular class of problems for decades while relying on a restricted set of working assumptions, even when alternative assumptions could yield better predictions. Second, organizing an open prediction competition may broaden the set of assumptions considered by the community and facilitate the discovery of more effective models. Evidence consistent with these predictions comes from the choice prediction competition conducted in 2015 and summarized by Erev et al. in 2017, referred to as CPC15 [25]. The competition focused on decisions under risk and uncertainty, a class of choice problems that behavioral decision researchers had studied extensively for more than 70 years. The dominant models in this literature are variants of prospect theory, which was introduced by Kahneman and Tversky in one of the most highly cited and influential papers in social sciences [27]. (As demonstrated by Wakker [28], some of these assumptions are also consistent with the foundations of expected utility theory set by von Neumann & Morgenstern [29].)

Erev and colleagues [25] ensured that the space of tasks they examined included tasks exhibiting the behavioral regularities captured by prospect theory. Specifically, their training data set included replications of the phenomena that motivated the presentation of prospect theory: the certainty effect, the reflection effect, loss aversion, and overweighting of rare events. In addition, the space included tasks that replicate known effects of experience and ambiguity.

Prospect theory builds on several key assumptions. First is the implicit working assumption that decision makers read, understand, and believe the description of the payoff distributions [30]. In addition, it assumes that decision makers consider each choice task and each prospect in isolation. Specifically, the attractiveness of each prospect is computed as a weighted average of the subjective value of each possible payoff. The weights reflect a subjective function of the objective probabilities. Some submissions to the competition built on these common assumptions, used them to derive initial predictions, and then applied machine learning to refine the predictions.

The results of the competition revealed that efforts to build on the assumptions underlying prospect theory were not successful. All the top-performing models built on a very different set of assumptions: those underlying the “Best Estimate and Sampling Tools” (BEAST) model. One important difference between the assumptions of BEAST and those of prospect theory concerns how decision makers are assumed to treat the description of the payoff distribution. Unlike prospect theory, BEAST assumes that decision makers doubt the accuracy of the descriptions. They behave as if they use the description to recall past experiences with similar descriptions and try to select the strategy that led to the best outcomes in similar situations.

Plonsky and colleagues [26] organized a second competition (CPC18) that extended the first CPC15 competition and evaluated the robustness of its results. In CPC18, the organizers considered a wider range of choice tasks, and competitors could use the results of CPC15 as a basis for refining their models. The results reinforced the CPC15 findings. The winning model built on BEAST and improved its predictions using a machine-learning (gradient boosting) refinement.

## 4. Prediction Accuracy, Understanding, and Skills

The choice prediction competitions described above challenge the assumptions underlying prospect theory, demonstrating that models based on different assumptions achieve substantially better predictive performance. Yet these competitions have had only a limited impact on the popularity of prospect theory: for example, the number of Google Scholar citations of the original prospect theory papers has continued to increase since 2017 (at least through 2025). One possible explanation is that prospect theory and related models were developed primarily to facilitate understanding rather than to maximize predictive accuracy. From this perspective, poor predictive performance does not necessarily diminish these models’ scientific value. This argument has merit, but it raises an important question. If behavioral scientists seek both to understand and predict human behavior, it is not clear what can be learnt from a deep understanding of models that consistently produce poor predictions.

At the same time, predictive success alone is not sufficient. If the winning model in a prediction competition is a highly complex machine-learning algorithm, its contribution to understanding the underlying psychological processes may be limited. One way to address this concern is to organize complementary competitions that reward explanatory value. For example, competitors could be asked to submit a one-page description of their model, and the extent to which the model is understood could then be evaluated empirically. Under one such method, independent judges would be asked to predict the model’s predictions for new problems based solely on its description. Models would score highest when judges can accurately anticipate the models’ predictions and when those predictions accurately forecast human behavior. This approach would reward models that combine predictive accuracy with interpretability.

Our preferred explanation for the continued popularity of prospect theory is that scientific progress often resembles the touch-typing example introduced in the opening section of this paper. That is, a simple demonstration that a superior approach exists, or even recognizing its advantages, is rarely sufficient to induce change. Researchers who have developed expertise in estimating the parameters of models built on a particular set of assumptions may gain little immediate benefit from adopting an entirely different modeling framework. Benefiting from the advantages of the new approach requires acquiring new concepts, methods, and technical skills. Transition is costly. This observation underscores the importance of developing institutional mechanisms to reduce reliance on restrictive working assumptions and to facilitate the adoption of effective and better alternatives.

## 5. Costs and Benefits for Organizers and Editors

Recognizing that prediction competitions can advance science may not be sufficient to motivate researchers to organize them. Organizing a competition requires substantial effort, and the expected benefits to the organizers may not justify these costs in the short term. However, in addition to scientific advancement, prediction competitions hold promise for early-career researchers. In their perspective article, Haubrock et al. [13] call for extensive institutional change, including, among other things, changes to review procedures and evaluation criteria. The use of choice prediction competitions is an example of a change of this type. We suggest that journal editors can help and facilitate this change. For instance, when evaluating papers that introduce refinements to existing models, or new approaches, editors could consider an additional type of decision: “acceptance conditional on the organization of an appropriate prediction competition.” That is, the paper would be accepted as Part 1 of “a registered report” [31] and would also lead to a competition that would further encourage the exploration of risky research. Under this option, the authors would secure publication and would organize an open competition in which other researchers are invited to develop and evaluate alternative models for the same prediction tasks. Such a minimal policy would facilitate systematic comparison of competing models, encourage advances in predictive performance, and increase the scientific value of published work. Increasing the benefits associated with organizing prediction competitions while creating opportunities for alternative approaches to be considered could help align the incentives of authors, editors, journals, and the broader scientific community.

## 6. Summary

Previous research suggests that traditional academic review processes reward adherence to prevailing assumptions, potentially slowing scientific progress. This paper illustrates an underlying mechanism with a simple sequential conformity game and argues that complementing conventional peer review with prediction competitions could mitigate the problem. Prediction competitions can transform the incentive structure from one in which the costs of conformity increase with group size to one in which social interaction accelerates convergence toward better strategies.

## Figures and Tables

**Figure 1:**
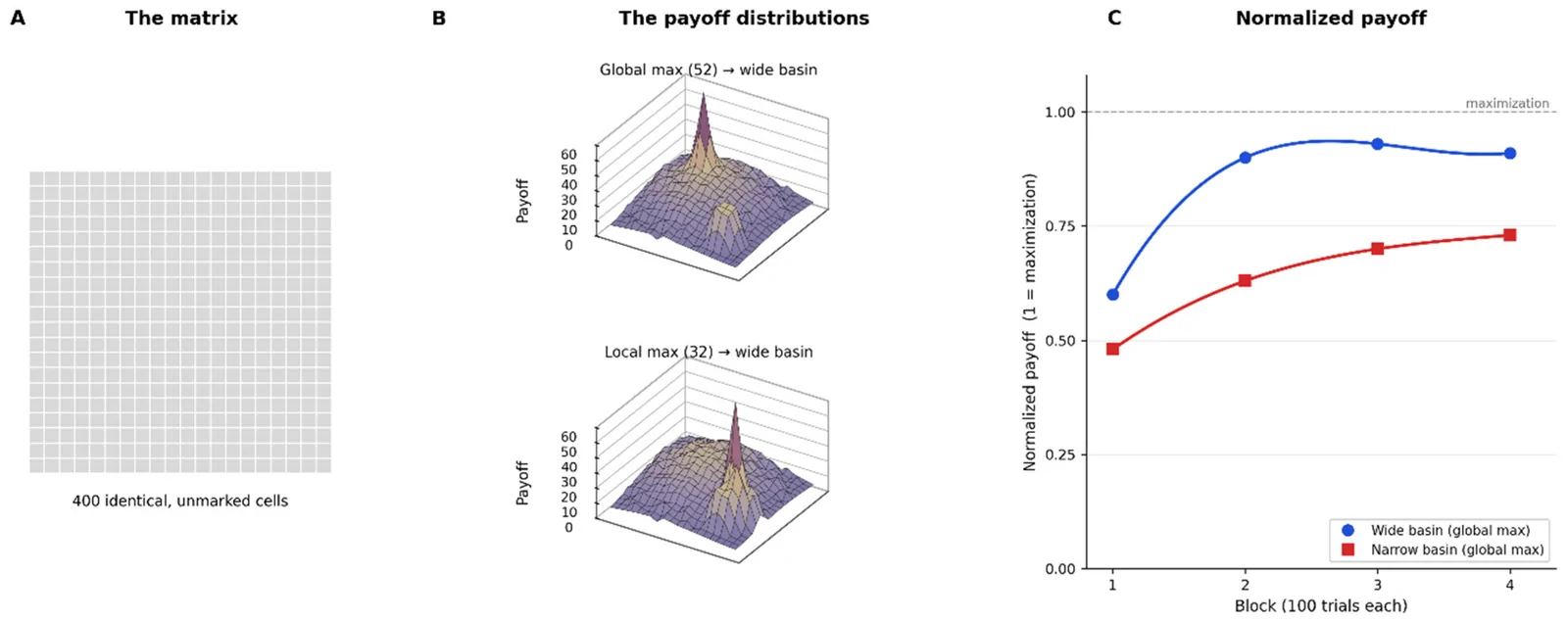
A demonstration of the descriptive value of the hill climbing hypothesis. Note: On each trial, decision makers picked one cell of a matrix with 400 unmarked cells. The hidden distributions of payoffs are depicted in the middle (brown implies high payoffs, and blue implies low payoffs). The right-hand panel presents the normalized payoff (where 1 implies maximization) in four blocks of 100 trials. The upper (blue) line represents the condition in which the global maximum had a wide basin (**top graph**), and the lower (red) line represents the condition with a narrow basin of attraction (**bottom graph**).

**Figure 2:**
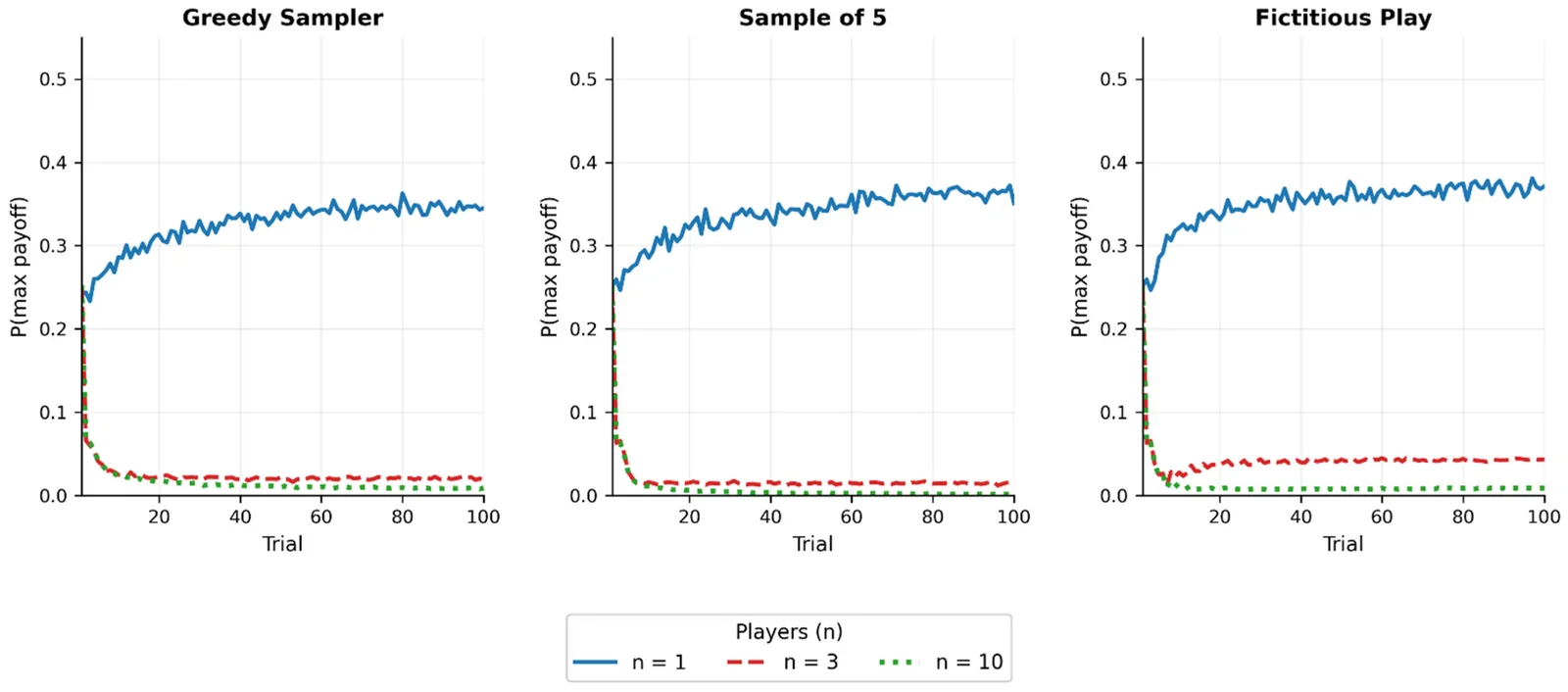
Predicted probability of the maximal payoff in a repeated play of the current sequential convention game provided by three models. Note: The titles Greedy Sampler, Sample of 5, and Fictitious Play each represent a different learning model. The predictions of the different models are consistent, indicating an advantage for individual choice over coordinated choice in the sequential conformity game. The specific learning rule for each model is described in Appendix A.

**Figure 3:**
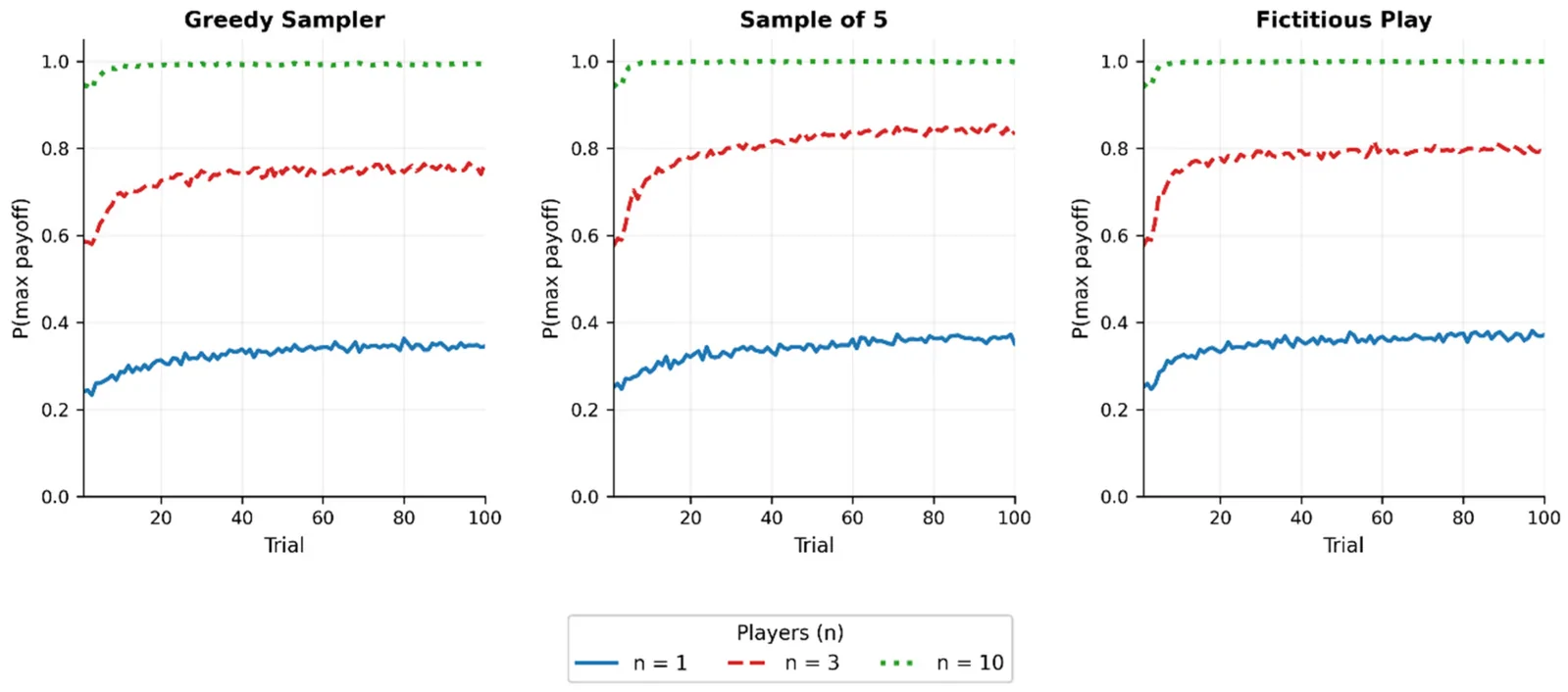
Predicted probability of the maximal payoff in a repeated play of the current competition game for *n* = 1, 3, or 10. Note: For this variant of the sequential conformity game, individual choice is outperformed by competition in groups of 3 players, and the best (maximizing) performance is achieved with larger groups. Each panel presents the simulated prediction of one of the three models used for the original sequential conformity game. The three models are described in Appendix A.

## Data Availability

The original contributions presented in this study are included in the article. Further inquiries can be directed to the corresponding author.

## Appendix A

### Appendix A.1. The Greedy Sampler

The Greedy Sampler model [32] was originally proposed to address repeated decisions based on full feedback. The current analysis uses a generalization [33] to situations where feedback is limited to the payoff from the selected option. It assumes that each choice is made based on old experiences that occurred before the beginning of the current choice task (e.g., experience with similar descriptions), and “new experiences” derived from observing the outcomes of others. The probability of reliance on old experiences after observing at least t past outcomes from each option is $\delta_{i}^{\left(\frac{t-1}{t}\right)}$ where δ_i_ is a free parameter of player *i*.

When player *i* relies on new experiences, the player samples with replacement κ_i_ (a second free parameter) of the observed outcomes from each option and selects the class that provided the best average payoff in the sample. In the current setting, when the players do not receive a prior description of the game, reliance on past experience is assumed to trigger random choice. In addition, the model assumes that the very first payoff is recorded as an experience with both options.

The predictions were derived using the joint distribution of κ_i_ and δ_i_ estimated by Erev and colleagues [32] using a constrained maximum likelihood method on the results of 87 distinct binary choice tasks. Table A1 presents this distribution.

**Table A1:** The estimated distribution of the Greedy Sampler parameters.

	*δ_i_*							
***κ_i_***	**0.01**	**0.05**	**0.1**	**0.2**	**0.3**	**0.4**	**0.7**	**1.0**
**1**	9.3	3.4	2.2	2.2	1.7	2.2	2.8	9.5
**2**	3.3	1.6	0.9	1.1	0.6	1.0	1.3	
**3**	1.5	1.2	0.8	0.4	0.4	0.8	0.5	
**4**	0.9	0.4	0.4	0.3	0.2	0.4	0.5	
**5**	1.0	0.4	0.4	0.5	0.3	0.3	0.5	
**6**	0.5	0.3	0.3	0.1	0.2	0.3	0.5	
**7**	0.6	0.4	0.3	0.3	0.2	0.2	0.2	
**8**	0.7	0.2	0.2	0.2	0.2	0.2	0.4	
**9**	1.1	0.4	0.4	0.4	0.2	0.4	0.3	
**12**	0.5	0.2	0.3	0.2	0.2	0.5	0.6	
**15**	0.6	0.4	0.2	0.1	0.1	0.2	0.2	
**20**	1.1	0.4	0.2	0.5	0.2	0.5	0.5	
**25**	1.0	0.5	0.4	0.4	0.3	0.2	0.4	
**30**	1.9	0.9	0.8	0.7	0.4	0.8	0.7	
**Very large**	3.2	2.4	1.8	2.1	2.9	2.7	3.0	

Note. The value of the parameters are marked in Bold. Cells indicate the percentages of cases. When the value of *κ_i_* is “Very large”, all past experiences were equally weighted, as expected when the sample size is very large (→ ∞).

### Appendix A.2. The Sample of 5 Model

The Sample of 5 model [34] is like the Greedy Sampler with the constraint κ_i_ = 5, and δ_i_ → 0.

### Appendix A.3. The Fictitious Play Model

The fictitious play model [35] is like the Greedy Sampler with the constraints:

κ_i_ → ∞, and δ_i_ → 0.

Notice that in the current setting, the prediction on fictitious play is similar to the prediction of basic reinforcement learning and the natural implementation of a Bayesian learning model.
